# Risk Perceptions and Safety Cultures in the Handling of Nanomaterials in Academia and Industry

**DOI:** 10.1093/annweh/wxaa022

**Published:** 2020-03-10

**Authors:** Marie Louise Kirkegaard, Pete Kines, Katharina Christiane Jeschke, Keld Alstrup Jensen

**Affiliations:** 1 National Research Centre for the Working Environment, Copenhagen, Denmark; 2 Technical University of Denmark, Department of Technology, Management and Economics, Lyngby, Denmark; 3 Department of Organization, Copenhagen Business School, Frederiksberg, Denmark

**Keywords:** compliance, hierarchy of prevention, induction, nanotechnology, occupation safety and health, precautionary principle, risk comprehension, safety data sheets

## Abstract

**Objectives:**

Work and research with nanomaterials (NMs) has primarily focused on innovation, toxicity, governance, safety management tools, and public perceptions. The aim of this study was to identify academia and industry occupational safety and health (OSH) managers’ perceptions and handling of NMs, in relation to safety culture.

**Methods:**

Semistructured interviews were carried out with OSH managers at six academic institutions and six industrial companies. The interview statements were coded into five topics regarding NMs: risk comprehension, information gathering, actions, communication, and compliance. The statements were then coded according to a five-step safety culture maturity model reflecting increasing occupational safety maturity from passive, to reactive, active, proactive, and exemplary occupational safety.

**Results:**

The safety culture maturity of the academic institutions were primarily *active* and *proactive*, whereas the industry group were primarily *active* and *reactive*. None of the statements were rated as *exemplary*, with the majority reflecting an *active* safety culture. The topics varied from a *passive* approach of having no focus on NMs and regarding risks as a part of the job, to applying proactive measures in the design, production, application, and waste management phases. Communication and introduction to OSH issues regarding NMs as well as compliance provided challenges in both academia and industry, given the increasing cultural and linguistic diversity of students/staff and employees. Workplace leaders played a crucial role in establishing a legitimate approach to working safely with NMs, however, the currently available OSH information for NMs were described as insufficient, impractical, and inaccessible. There was an embedded problem in solely relying on safety data sheets, which were often not nanospecific, as this may have led to underprotection.

**Conclusions:**

There is a need for more structured, up-to-date, easily accessible, and user-friendly tools and information regarding toxicity and threshold limit values, relevant OSH promotion information, legislation, and other rules. The study underscores the need for politicians and engineers to collaborate with communication experts and both natural and social scientists in effectively framing information on NMs. Such a collaboration should allow for flexible deployment of multilevel and integrated safety culture initiatives to support sustainable nanotechnology and operational excellence.

## Introduction

Nanotechnology is cutting-edge science, and a fast-growing applied technology (Khan and Asmatulu, 2013). It is one of six key enabling technologies that can lead to sustainable economic and social development covering almost all economic sectors on a global scale (EUON, 2019; European-Commission, 2019). Nanomaterials (NMs) are natural, engineered, and process-generated materials containing over 50% nanosized particles (1–100 nm; 1 × 10^−9^ m) (EU, 2018b). Due to their smaller size, higher specific surface area, and changes in properties (e.g. higher reactivity), NMs can be more hazardous than larger sized particles of the same composition (Berges *et al.*, 2014; Stone *et al.*, 2017; EUON, 2019).

Nanoparticles may enter the body through inhalation, ingestion, dermal contact, ocular and skin penetration, etc. (Oberdörster *et al.*, 2005; Ryman-Rasmussen *et al.*, 2006). Inhalation of nanosized particles has primarily been linked to pulmonary toxicity, cancer, cardiovascular disease, and reproductive toxicity (Savolainen, 2014; Hougaard *et al.*, 2015; Stone *et al.*, 2017) (see also Fadeel *et al.*, 2017). The rate of nanotechnology development and implementation in this transdisciplinary field have, however, outpaced the rate of generating knowledge of its potential implications (Grieger *et al.*, 2019; Porcari *et al.*, 2019). Unlike the experience with previous risks such as nuclear energy (radiation) and asbestos, the potential applications of nanotechnology are much greater and widespread (Geraci, 2017) with it being used in e.g. construction, composite materials, electronics, energy, healthcare, pharmaceuticals, clothing, and food (Nabhani *et al.*, 2012; Read *et al.*, 2014; Foster, 2017). The implications of NMs on people, animals, and the environment may last throughout the life cycle (value chain) of NMs, from research and design, manufacturing, handling, application/use, to recycling and disposal (Read *et al.*, 2014; Bekker *et al.*, 2015; Fadeel *et al.*, 2017; Khan and Asmatulu, 2017; Trybula and Newberry, 2017).

Students, technicians, researchers, and industrial workers are the first to be subjected to the risks of NMs (Koiranen *et al.*, 2017; Kuijpers *et al.*, 2017; Fonseca *et al.*, 2018). Managing NM risks may mean that: ‘*Many of the precepts of safety and health have to be re-examined… to support safe and responsible development of nanotechnology*’ (Geraci, 2017, p. V). For good risk governance, the ‘precautionary principle’ has been proposed to be systematically enforced in all research, development, and industrial production and use of NMs. According to the principle, appropriate precautionary measures should be taken even when the cause and effect relationships are not fully established scientifically (COMEST, 2005; European-Commission, 2008). A number of guidelines and tools exist for occupational safety and health (OSH) management of NMs, however, recent reviews still find that the efficacy data of risk management measures for NMs are limited and inconclusive (Goede *et al.*, 2018), and that further improvements are needed in nanorisk governance (Grieger *et al.*, 2019; Porcari *et al.*, 2019).

Risk management approaches have to date often been based on the hierarchy of controls involving: elimination, substitution/modification, containment/isolation, technical/engineering controls, organizational/administrative controls, personal protective equipment (PPE), and simple instruction (Sayes, 2017; Tate and Hernandez, 2017). However, in a recent survey (Nørskov *et al.*, 2017) of over 7000 companies potentially working with NMs in Denmark, 26% replied that they did not have the necessary knowledge about NMs, and few (16%) knew of the NM guidelines provided by governmental authorities. Noteworthy, 29% of the companies did not perform a chemical risk assessment as required by law, and 37% did not provide specific introduction and instruction to the employees potentially exposed to NMs. Nørskov *et al.* estimated that over 15 500 workers in Denmark were potentially at risk of occupational exposure to NMs.

A five-step ‘safety culture maturity model’ (Table 2) has been proposed in analyzing how groups approach occupational safety, e.g. from ignoring risks to more exemplary approaches (Parker *et al.*, 2006; Hudson, 2007; Cooper, 2018; Cunningham and Jacobson, 2018). Administrators, practitioners, and researchers have adopted and adapted the term ‘safety culture’ (McGinn, 2010; NRC, 2011; ACS, 2012; Staehle *et al.*, 2016; Stuart and McEwen, 2016; Cunningham and Jacobson, 2018) and the maturity model to describe workplace and laboratory safety (McGarry *et al.*, 2013; TFACLSSU, 2014). However, the model has not been used in analyzing OSH perceptions and handling of NMs in academic and industrial settings. Such an approach could help identify OSH managers’ understandings of NM risks, particularly when NMs are not a central part of a company’s risk management activities. It could also further the understanding of where industry and academic stakeholders obtain their information, which actions they take, how they communicate, and how they perceive their compliance.

The aim of this paper is to identify academia and industry OSH managers’ perceptions and handling of NMs, and to put this in relation to the five steps of the ‘safety culture maturity’ model.

## Methods

Semistructured in-depth interviews were conducted with Danish and Swedish OSH leaders/managers from six academic institutes and six industrial companies that were known to work with manufactured NMs. The institutes and companies were identified through heterogeneous (maximum variation) sampling (Suri, 2011; Palinkas *et al.*, 2015) to obtain a variety of research institutes (e.g. traditional and within different technical fields) and companies (e.g. paints, adhesives, spays, waste handling, and industry equipment development and testing) with diverse characteristics to describe key topics in the sectors (Saunders *et al.*, 2015). An additional 11 other companies and 3 academic institutions were also approached, including pharmaceutical and biotechnology companies, but they either declined to participate or did not reply. All participating companies and five of the academic institutes were located in Denmark. One academic institute was recruited from Sweden, as there were too few academic institutes working with NMs in Denmark.

The interviews were completed in 2016–2017, before the REACH regulation was changed to include specific registration of NMs as substances on nanoform from January 2020 (EU, 2018a). However, we consider that changes had not occurred in the interviewees’ awareness and risk management practices at the time of the interviews.

Interviewees were OSH managers from different organizational levels within the institutes and companies. Informed consent was obtained from the interviewees, and their participation was not compensated. One or two of the authors conducted the interviews, and all authors participated at least once. The interviews were recorded, and transcribed as detailed resumes, with key citations transcribed verbatim (Halcomb and Davidson, 2006; Kvale and Brinkmann, 2009; Richards, 2014). The interview statements were coded using Nvivo 12 (Saunders *et al.*, 2015). First, the interview statement was analyzed by the main author, and coded into one or more of five nano OSH topics—Table 1. Secondly, the first and second authors discussed and refined the results. Thirdly, the two of the authors coded the statements according to the safety maturity model (Table 2).

**Table 1.** Main questions in the semistructured interview guide: Occupational safety and health (OSH) topics regarding nanomaterials (NMs). Interviewees were also asked to provide examples for each question.

Risk comprehension
• How do you understand the term ‘nanomaterials’ (NM)?
• How do you understand the term ‘risk’?
Information gathering
• How do you obtain information on how to deal with NM? …how to work with NM?
• How do feel about the quantity and quality of information you gather regarding NM and OSH risks? Sufficient?
Actions
• How are OSH risks with NM identified and dealt with in general? On a daily basis?
• To what degree are prevention principles applied? (Hierarchy of controls) And how?
Communication
• How are risks and risk management strategies and initiatives communicated to employees/students and collaborators?
Compliance
• How motivated is the company/institute toward compliance with OSH regulations and guidelines with NM?
• How motivated are students/workers/leaders in complying with OSH regulations and guidelines with NM?

**Table 2.** Safety culture maturity levels—examples of five topics.

Passive	Communication	• Low information flow
		• Fear of reprisals
	Cooperation	• Low degree of cooperation
	Benchmarking	• ‘Getting away with it’, scapegoats
	Safety practices	• Accidents are a part of the job, not preventable
		• Safety is an expense
	Leadership	• Disinterest in regulation
		• Sanctioned deviance from safe practice
Reactive	Communication	• No follow-up
	Cooperation	• Control of the individual—not the process
	Benchmarking	• Prices compete with safety
		• Safety is first taken seriously after an incident
	Safety practices	• No systematic approach
		• Focus on operational factors
	Leadership	• Audits are punishments
		• Reacting to demands from regulators and incidents
Active	Communication	• Through standardized channels and procedures
		• Remains local
		• Standard safety training
	Cooperation	• Slight cooperation and trust
	Benchmarking	• Internal benchmarking on measurable factors
	Safety practices	• Incidents lead to quick fixes
		• Some safety initiatives
		• The underlying problems are often ignored
	Leadership	• Policies and procedures are rarely implemented or enforced
Proactive	Communication	• Information is shared in the organization
		• Systematic follow-up and evaluation
	Cooperation	• Sincere dialog and partnership
	Benchmarking	• With others in the sector
		• They want to be the best
	Safety practices	• Focus on the process not the outcome
		• Incidents are explored in-depth
	Leadership	• Management encourages safe behavior
		• Audits are seen as a help
Exemplary	Communication	• Information is also relevant, timely and suitable
		• Learning from incidents
	Cooperation	• High degree of cooperation
		• Empowerment and participation on all levels
	Benchmarking	• Benchmarking across sectors
	Safety practices	• Safety practice is embodied through design
		• New ideas are implemented and assimilated quickly
	Leadership	• Visible and credible safety leadership
		• Open reporting culture

## Results

All 12 cases within industry and academia worked with manufactured NMs in slurry and/or powder form, but only 2 worked with NMs as their primary activity. The industry cases handled intermediates and final products with high volume pigments, fillers and additives to paints and adhesives, polymers in plastics and master batches, and a wide range of NMs in waste handling. The academic cases involved research regarding nanotechnology, energy, the release, exposure, and (eco)toxicological effects of NMs and NM-enabled products. The academic departments themselves were small (under 50 staff/students), but were part of larger institutions. The industry cases varied from medium to large sized enterprises with over 1000 employees. The academic institutes worked primarily and intermittently with µg to 100 g amounts of NMs at a time, whereas industry regularly worked with larger quantities measured in hundreds of kilograms.

Ten to 40 statements were coded from each of the 12 cases, and none of the statements met the coding requirements reflecting the highest step of *exemplary* safety culture. *Passive* statements were rare, while *active* were the most common (Table 3).

**Table 3.** Occupational safety and health topics regarding nanomaterials: number of interview statements in the interviewed organizations and their related safety culture level.

	Risk comprehension	Information	Actions	Communication	Compliance
Academia					
Passive	0	0	0	0	0
Reactive	0	5	10	3	9
Active	18	20	31	18	17
Proactive	6	5	10	8	9
Exemplary	0	0	0	0	0
Industry					
Passive	3	0	3	0	0
Reactive	7	15	22	6	12
Active	11	17	33	3	7
Proactive	2	4	13	4	1
Exemplary	0	0	0	0	0

### Risk comprehension

Statements from interviewees regarding risk comprehension in both *academia* and *industry* focused on the safe handling and potential adverse health effects of NMs. In *industry*, the focus was on long-term health risks. They typically perceived NM risks as a part of the job, and not as something employees should be afraid of. The statements from interviewees in *academia* more uniformly expressed that there were no reasons to worry, as long as employees handled NMs properly and with precautions:

Nano-risks are ‘actually the same as with any other OSH risks… if exposed, you can develop a disease… but it’s a matter of exposure, and an attempt to prevent exposure.’ (Academia 1)

The passive statements did not focus on the risks of NMs, whereas the *reactive* statements focused on the legal requirements for materials with the same chemical composition, and that nanoparticles had always existed. They did not express and understanding of when and where NMs could be dangerous. Some *active* statements focused on the safety management systems in place to assess risks. Safety was in some cases managed with focus primarily on legislative demands. The employees were perceived to have the primary risks—not knowing the OSH risks of the product, nor if the OSH precautions were sufficient. The perception of risk was affected by the form of the NMs (slurry versus powder, with powder as the greatest risk), as well as the frequency and duration of work with NMs. They often linked the minimization of risks to planning and application of precautionary measures. Some perceived NM safety as a strategic initiative, yet often as minor and vague, and only as secondary to economic factors. The *proactive* statements were in regard to effectively dealing with safety, and reflected more the precautionary principle. They tried to avoid any contact or spill of NMs when handling them, and in one case, they had stopped all work with NMs, when they realized that there were potential hazards, until new procedures were in place. Another described having marked all instruments used for NMs, which allowed the employees to choose different instruments.

### Information

In both *academia* and *industry*, relevant and sufficient data were perceived as very difficult to obtain, and complex technical data as difficult to use.

‘We started the company as a bunch of amateurs… the Internet was extremely helpful… but we didn’t have any of the right processes, as none of us had the right background… and because it is a start-up business, we couldn’t afford to buy expert advice until relatively late in the process.’ (Company 1)

This was the case even for highly educated and knowledgeable employees. There was uncertainty about e.g. how to set threshold values, and how to react if these values were exceeded. Many relied on ‘safety data sheets’ (SDSs) provided by suppliers, where the sheets were rarely nanospecific. Most of the cases in *academia* used the same database where they could log their NMs, identify risks, and find information about how to handle them. Some institutes had joined a nanonetwork, to share knowledge and strive for mutual understandings for handling NMs. Cases from *industry* would contact suppliers, consultants, trade associations, or search for additional information online.

None of the information statements were rated as *passive.* The *reactive* statements focused on acquiring information from the suppliers or in the internet ‘jungle’, and sometimes reverted to using traditional PPE. They described using organizational tools to protect the facilities, and not necessarily the employees. The *active* statements had the fundamental position that risks should be minimized, and that guidelines should support the work done, particularly concerning the employees’ exposure to unknown risks. The interviewees found the SDSs to be insufficient, requiring further information from suppliers. The standard operating procedures required the employees to plan the entire work process to allow for the sharing of knowledge. Several of the interviewees kept abreast of legislative changes, and used a risk-banding program to ensure that the workplace instructions were sufficient. However, the work with finding relevant information on safe procedures often stagnated after the initial search was satisfied. The *proactive* statements focused particularly on keeping up-to-date with legislation and on how dangerous materials reacted under different conditions, e.g. aging and weathering. One interviewee described that their procedures for working with NMs were certified by an external authority, and new work with NMs was not begun before information on the risks and precautionary measures were established.

### Actions

Both groups described management commitment and support as an essential factor for creating ownership toward OSH and handling risks. Interviewees from *academia* considered procedures for handling NMs as good, if they were useful, easily accessible, and simple. The statements from *industry* varied greatly, with some focusing on administrative and technical initiatives, and others on workplace instructions. The role of leadership commitment to OSH is reflected in the interviews:

‘…there’s been a positive development (use of PPE)… and there’s no doubt that if it’s to succeed, then it has to come from higher up (in the organization)… right up from the top, and it has to permeate the culture.’ (Company 2)

The *passive* statements were from interviewees who did not have a chemical workplace assessment nor any precautionary measures for handling NMs, as they thought they worked with too small amounts (e.g. a few 100 g). The *reactive* statements were from interviewees who trusted the quality of the labels provided by suppliers, even though they described them as insufficient. They did not have any particular strategy for working with NMs nor for obtaining further information, and often did not treat NMs any differently than other materials (e.g. chemicals). The *active* statements focused on daily planning, with examples of risk prevention and primarily using PPE, but also attempts of using substitutions, as well as administrative and technical measures. Employees were instructed in handling NMs, and there was open dialog between them and management on responsibilities. Some cases had measured the concentrations of nanoparticles during certain procedures, to ensure that the exposure level was low. Some initiated plans to improve the lack of measures, but these did not include information on ‘when’ to implement the improvements. In the *proactive* statements, risks were eliminated by e.g. limiting employee contact with NMs, transferring materials safely between different processes, dedicating and modifying work sites and/or equipment for handling NMs, having external experts evaluating the process, having protocols for the daily procedures, and training employees specifically in how to handle NMs.

### Communication

In *academia*, a thorough introduction involved OSH nationally (e.g. regulations) and locally, and the employers’ expectations to the employees. The interviewees found it challenging to communicate across different cultural and linguistic backgrounds, and the variable understandings of OSH:

‘Our risk assessments are targeted to both the project and the individual, because people are so different—culturally. We’ve created a simple list to try to assess their understanding of the risks.’ (Academia 2)

In the *industry* group, companies targeted their introductions toward the specific work task. There were no statements rated as passive. The *reactive* statements revealed a limited form of general introduction and peer-to-peer instruction. Employees were informed if specific changes were made, but employees were otherwise to find relevant information themselves. There were no follow-ups after the introductions, and the organizations introduced changes in procedures primarily via SDS, or by posting minutes from the OSH organization on the intranet, where employees could read them. If they offered OSH courses, they were only for managers. The *active* statements reflected keeping employees updated about changes, and telling them where to obtain more knowledge if needed. Guidelines were adapted and included technical information that was relevant for specific work tasks. In most cases, new employees were provided with both a general OSH introduction and an introduction to the specific tasks, including being offered safety education and training courses. The *proactive* statements had a strong focus on educating employees through training, retraining, follow-up and the possibility for certification. There was also a stronger focus on sharing information continuously across different work functions, and adapting materials to specific groups. An introduction included discussions with new employees about their previous experiences—with particular attention to overly confident employees and employees apprehensive of asking questions. Focus was on creating awareness of the potential risks and on stopping processes if further reflection was required. The interviewees thought that the top leaders had a strong focus on increasing the level of safety, and they included all the employees in a discussion about potential hazards and about how to improve safety in upcoming projects.

### Compliance

In *academia* compliance was often seen as depended on e.g. the level of seniority (younger were perceived as working safer than older) and function (lab assistants/technicians were perceived as working safer than researchers). They attributed noncompliance to a low managerial focus on OSH, the perceived ‘visibility’ of the OSH organization, and the availability of and easy access to PPEs.

Generally, interviewees in both *academia* and *industry* found the level of compliance to be increasing over time, which in some cases was attributed to a change in leadership:

‘We were previously not so good at using PPE, but have gotten better… maybe because we got a new factory manager, who reminds us to use them, and that we as leaders have also been encouraging their use.’ (Company 3)

Compliance in *industry* varied with some describing a zero-tolerance for not complying with OSH rules. In the ‘*reactive*’ statements, the interviewees described that the managerial and senior employees’ approach to safety influenced the other employees’ attitudes. Managers did not perceive all tasks as hazardous, and it was thus difficult to persuade employees to use PPEs and read instructions. The interviewees’ attempts to improve OSH were often seen as hampering work progress. The *active* statements described compliance as closely connected to the attitudes and actions of management, and whether or not they enforced rules. Several of the interviewees attempted to ensure that anyone working near potential risks also complied with PPE rules, even though their own work tasks did not prescribe PPE. Employees were encouraged to promote compliance e.g. if they saw someone not wearing appropriate PPE. The OSH organization evaluated compliance to see if their initiatives worked. The *proactive* statements focused on formalizing guidelines with inputs from the employees or joint forums, and then having management announce them, e.g. a zero-tolerance. The OSH organization was to consistently inspire and ensure employee compliance, responsibly awareness and engagement. Safety was included as a major consideration in connection to acquisition, rebuilding, and relocating.

## Discussion

About two-thirds of the statements on ‘risk comprehension’ were rated as *active* or *proactive*. Previous studies have shown that industries working with NMs are confident in their ability to understand risks (Becker, 2013). Díaz-Soler *et al.* (2017) showed that knowledge of risks and control measures increased with the number of different types of NMs used. Regardless of how institutions and companies obtain information on NMs, the source’s ‘framing’ of the NM information, as well as the recipients’ ‘cultural cognitions’ are important to consider when communicating about NMs (Kahan *et al.*, 2009). Cultural cognition deals with how individuals and groups interpret information about risks (to NMs), and that they often interpret risks in a way that follows their own cultural and political values, e.g. being risk critical toward NMs. The messages and credibility of a source may be interpreted quite differently by e.g. industry professionals, who are largely influenced by the information. There is a need to take both credibility and culture into consideration when providing ‘balanced’ information from sources, whose values the recipients identify with.

Approximately one-third of the statements in the current study were rated as *reactive* concerning ‘information’. Previous studies have shown that NM OSH information, guidance and regulation are not reaching industry (Conti *et al.*, 2008; Engeman *et al.*, 2012) nor academia (Díaz-Soler *et al.*, 2017). Nørskov *et al.* (2017) recently showed that many companies did not actively seek OSH information related to challenges with NMs. If they did, they mainly looked to the national OSH authorities (29%), producers (14%), internal (14%) or external experts (11%), OSH sector councils (8%), suppliers (7%), and various other sources (33%)—with variations between low, medium, and high-tech companies. Another study (Helland *et al.*, 2008a,b) showed that only a few companies working with NMs had standardized criteria or procedures for raw material substitution, should their risk assessment reveal any potential risks.

In the current study, 30% of the ‘action’ statements were rated as either *reactive* or *passive*, with descriptions of a lack of procedures and strategies to handle NMs. Previous studies have however shown that research institutes are less prone to have NM-specific OSH programs than companies (Conti *et al.*, 2008). Likewise, NM risks are often ignored, and hygienic and health monitoring were found non-existent in research facilities (Díaz-Soler *et al.*, 2017). A large multinational study showed that companies often use ‘general chemicals risk management’ instead of NM-specific prevention (Iavicoli *et al.*, 2019). Moreover, OSH measures are more prevalent in organizations working with powder-based than liquid based applications (Schmid and Riediker, 2008). In addition, companies with nanospecific OSH programs rarely ensure that they cover all aspects of the life cycle of a product (from research and design to disposal) (Engeman *et al.*, 2012; Bekker *et al.*, 2015). In the Nørskov *et al.* (2017) survey, there were substantial variations between how different OSH measures were used both between and within different industrial sectors: 75% carried out a general risk evaluation, 34% applied substitution or work alteration initiatives, and the most commonly used technical or PPE exposure control strategies were ventilation (92%), gloves (88%), fume hoods and/or gloveboxes (56%), and respiratory devices (55%)—with variations between low, medium and high-tech companies. Iavicoli *et al.* (2019, companies) and Díaz-Soler *et al.* (2017, research facilities) also found that gloves (97 and 93%, respectively) as well as eye/face protection (91 and 73%, respectively) were the most common form of PPE, whereas respiratory protection was used to a lesser degree (53 and 55%, respectively).

The majority of the statements regarding ‘communication’ through e.g. introduction and instruction were rated as either *active* or *proactive*. This is somewhat reflective of the survey from Nørskov *et al.* (2017), who showed that on average 58% of the firms carried out nanospecific instructions, yet only 34% had particular guidelines for working with NMs, and only 27% of the employees received special training. Furthermore, the high-technology companies did not have a higher degree of introduction and instruction than the lower technology companies. Iavicoli *et al.* (2019) also found that companies often train informally—peer-to-peer. These results indicate that there is a general need to increase the amount and quality of introduction, instruction, and follow-up (induction or onboarding), and that such an increase would be needed industry wide. Focus is often on formal safety compliance (e.g. protocols and procedures) and ‘what’ to do, rather than informal safety compliance and ‘how’ to integrate it practically and usefully into daily work (McGarry *et al.*, 2013).

In the current study, the level of ‘compliance’ was perceived as directly related to the presence of strong managerial commitment to safety. A recent study in academic laboratories in general (including NMs) found that institutional commitment to OSH was inversely related to the risk of accidents/injuries (Salazar-Escoboza *et al.*, 2020). There is a growing interest in holistic/integrated safety culture approaches involving all organizational levels with both top-down (leader-based) and bottom-up (student/worker-based) initiatives (McGarry *et al.*, 2013), with integration in all operational functions (Freibott, 2014), and where regulations act as the foundation (floor) rather than the limits (ceiling) for safety practices (Stuart and McEwen, 2016). Similar to a zero-tolerance approach and a ‘vision zero’ strategy for safety (health and wellbeing) (Zwetsloot *et al.*, 2017), Freibott (2014) warns against safety management being dealt with as an ‘*isolated functional area by capsulated experts who focus on safety processes and regulations to drive compliance and best practices only*’ (p. 6). In line with ISO45001 OSH standard (ISO, 2018), it is critical that OSH be more effectively integrated in an organization’s business strategy, processes, and leadership, as well as ensuring employee influence.

Rather than a *reactive* approach to new technological development, as was seen in the previous century with asbestos, radiation, genetic modification, etc., the current focus on the safe and responsible development of nanotechnology is a rare example of a proactive approach in technological development (Geraci, 2017). The current study is an important methodological contribution to looking at safety culture in nanotechnology. The method of combining topics and levels of safety could advantageously be used in future studies.

## Future needs and implications for research and practice

The interviews revealed a number of needs including structured, up-to-date, easily accessible and user-friendly information regarding regulations, occupational exposure limits, SDSs, technical data sheets, and chemical workplace assessments and instructions. This reinforces the need for politicians and OSH professionals to collaborate with communication experts and both natural and social scientists in providing reliable and easily understandable, and useable information (Kahan *et al.*, 2009; Kuempel *et al.*, 2012). Collaboration is necessary to handle the OSH challenges of emerging technologies, as the individual problem-solving capacities of each stakeholder group is limited (Linkov *et al.*, 2018). In addition, the information would likely be more meaningful, and prevention control more effective, if they were supplemented with personal and contextual exposure monitoring in the academic and industry work settings. Health and hygiene monitoring may also be an effective addition to nanospecific risk assessments (Díaz-Soler *et al.*, 2017). The role of age and position in an organization (lab assistant, student, worker, manager, and leader) in terms of OSH compliance should be studied further (Salazar-Escoboza *et al.*, 2020).

There are no one-size-fits-all solutions, and future initiatives should be organized in consistent ways that allow for ‘flexible deployment’ of multilevel and integrated safety culture initiatives to support operational excellence (Freibott, 2014). In seeking ‘sustainable’ nanotechnology there is a need to balance applications and implications in meeting current and future needs and their human, social, technical, economic and environmental impacts (Yang and Miao, 2010). Initiatives need to be transferable, scalable and sustainable (Stuart and McEwen, 2016), as the application of nanotechnology continues to evolve and expand to other branches and countries. It is critical to adapt initiatives to local cultures, however current safety culture research is geographically skewed, and underrepresented in areas such as Africa, Oceania, and South America (van Nunen *et al.*, 2018).

Adapting safety culture initiatives needs to consider a number of crucial differences between academia and industry. Laboratories in academia, as opposed to industry, are often small scale, they operate under a more flat—decentralized hierarchy, they have a higher turnover, are culturally and linguistically more diverse, and are rarely audited by OSH authorities and professionals. This thus challenge the potential to affect and sustain cultural changes in safety (Marendaz *et al.*, 2011, 2013; McGarry *et al.*, 2013; TFACLSSU, 2014; Koiranen *et al.*, 2017).

Future studies could improve on some limitations met in this study. The study’s validity is limited to analytical generalizability, and combined with the small sample size, should not infer directly to the general population. However, the results obtained align with the results from recent, broader international surveys (Porcari *et al.*, 2019). Future studies could also take into account the differential OSH training and organizational positions of the interviewees, as well as the potential social desirability bias of responses. Finally, studies could explore in greater depth company actions to comply with regulations and policies, as there are often different mechanisms and tools by which work place safety can be ensured.

## Conclusion

Interview statements from both *academia* and *industry* reflected a predominantly *active* safety culture across the topics of NM risk comprehension, information, actions, communication, and compliance. There was great variation from the *passive* and *reactive level*, where safety concerning NMs were generally ignored; no information was sought; NMs were managed as if they were general chemicals; there were very little instructions, and a low level of managerial commitment to safety. At the *proactive* level, there was a focus on the precautionary principle, keeping up to date, eliminating risks, sharing information across the organization, and a cross-organizational focus on safety.

There will continue to be untested risks with NMs for several years to come and currently SDSs rarely consider NM risks. There is therefore still a need to combine the precautionary principle with the measures from the hierarchy of OSH prevention to achieve ‘sustainable’ nanotechnology. In governing risk processes of NMs it is recommended that systems take into account the variability in the level of safety between and within sectors. Safety considerations should be included in all stages of the life cycle of NMs, and there is a need for developing effective (hard and soft) safety introductions, instructions and follow-ups on NMs for new as well as experienced employees.
